# A cytoplasmic quaking I isoform regulates the hnRNP F/H-dependent alternative splicing pathway in myelinating glia

**DOI:** 10.1093/nar/gku353

**Published:** 2014-05-03

**Authors:** Mariana D. Mandler, Li Ku, Yue Feng

**Affiliations:** Department of Pharmacology, Emory University, Atlanta, GA 30329, USA

## Abstract

The selective RNA-binding protein quaking I (QKI) plays important roles in controlling alternative splicing (AS). Three QKI isoforms are broadly expressed, which display distinct nuclear-cytoplasmic distribution. However, molecular mechanisms by which QKI isoforms control AS, especially in distinct cell types, still remain elusive. The quakingviable (qk^v^) mutant mice carry deficiencies of all QKI isoforms in oligodendrocytes (OLs) and Schwann cells (SWCs), the myelinating glia of central and peripheral nervous system (CNS and PNS), respectively, resulting in severe dysregulation of AS. We found that the cytoplasmic isoform QKI-6 regulates AS of polyguanine (G-run)-containing transcripts in OLs and rescues aberrant AS in the qk^v^ mutant by repressing expression of two canonical splicing factors, heterologous nuclear ribonucleoproteins (hnRNPs) F and H. Moreover, we identified a broad spectrum of *in vivo* functional hnRNP F/H targets in OLs that contain conserved exons flanked by G-runs, many of which are dysregulated in the qk^v^ mutant. Interestingly, AS targets of the QKI-6-hnRNP F/H pathway in OLs are differentially affected in SWCs, suggesting that additional cell-type-specific factors modulate AS during CNS and PNS myelination. Together, our studies provide the first evidence that cytoplasmic QKI-6 acts upstream of hnRNP F/H, which forms a novel pathway to control AS in myelinating glia.

## INTRODUCTION

Alternative splicing (AS) allows production of multiple mRNAs from a single gene that often encode distinct protein isoforms to perform paradoxically opposite functions (1). More than 90% of mammalian genes are regulated by AS, which greatly increases proteomic diversity (2). Inclusion or exclusion of an alternative exon is achieved by interactions between canonical splicing factors and specific sequences in the pre-mRNA, which in turn control the recruitment of the splicing machinery (3,4). The majority of splicing factors are ubiquitously expressed (1), yet alternatively spliced mRNA isoforms are often differentially regulated during development and in different cell types (5,6). How generic splicing factors achieve differential regulation of AS is an intriguing question that remains largely unanswered. In oligodendrocytes (OLs) and Schwann cells (SWCs), two types of functionally related glial cells that form myelin membranes to enable nerve conductance in the central and peripheral nervous systems (CNS and PNS), respectively, numerous transcripts are subjected to regulation by AS during myelin development (7,8). Dysregulation of AS in OLs causes human neurological diseases, as seen in the aberrant AS of proteolipid protein (PLP) pre-mRNA in a familial form of Pelizaeus–Merzbacher disease (9). In addition, severe dysregulation of AS is caused by deficiency of the quaking I (QKI) RNA-binding protein in myelinating glia of the homozygous quakingviable (qk^v^/qk^v^) mutant mouse, represented by the pre-mRNAs of PLP and myelin associated glycoprotein (MAG) (10–12).

Three QKI protein isoforms are derived from AS, named QKI-5, 6 and 7 (8). All QKI isoforms share the N-terminal K-homology (KH) domain for binding RNA whereas the distinct C-termini determine isoform-specific nuclear-cytoplasmic distribution (7,13–14). QKI-5 predominantly localizes in the nucleus at steady state (14–16). In contrast, QKI-6 and QKI-7 are largely cytoplasmic (11,14,16–18). The nuclear isoform QKI-5 is expressed in most cell types, and was previously shown to regulate AS of pre-mRNA and control mRNA nuclear export, whereas the cytoplasmic isoforms QKI-6 and QKI-7 govern stability and translation of their bound mRNAs (8,10–11,16,18–22). Interestingly, QKI-6 is the most abundant isoform in myelinating glia (21,23). Although all QKI isoforms are reduced in OLs of the qk^v^/qk^v^ mutant (14,21), OL-specific expression of cytoplasmic QKI-6 alone rescues AS abnormalities in the qk^v^/qk^v^ brain without increasing nuclear QKI-5 levels (11). Thus, QKI-6 must control an undefined post-transcriptional cascade, which in turn governs AS during myelin development.

Heterologous nuclear ribonucleoproteins (hnRNPs) F and H are functionally orthologous splicing factors, both of which target polyguanine (G-run) sequences within or surrounding alternative exons in pre-mRNAs to regulate AS (24–27). During differentiation of OL progenitor cells (OPCs) and CNS myelination, both hnRNP F and H are markedly down-regulated, which in turn regulates AS of numerous pre-mRNAs (28), including the PLP pre-mRNA known to be affected by QKI deficiency (10,12). However, molecular mechanisms that regulate hnRNP F/H expression still remain elusive. Moreover, the functional AS targets of hnRNP F/H in myelin development are largely unknown.

We report that the cytoplasmic QKI-6 acts upstream of hnRNP F/H to regulate AS in myelinating glia. Deficiency of QKI in OLs and SWCs of the qk^v^/qk^v^ mutant mice results in aberrant over-production of both hnRNP F and hnRNP H. Such dysregulation of hnRNP F/H in OLs can be completely rescued by transgenic expression of the cytoplasmic QKI-6 alone. Furthermore, we identified a pool of functional hnRNP F/H targets *in vivo*, which are differentially affected in OLs and SWCs of the qk^v^/qk^v^ mutant mice. These findings reveal that cytoplasmic QKI-6 and hnRNP F/H form a novel regulatory pathway to control AS in myelinating glia.

## MATERIALS AND METHODS

### Animal colonies and treatment

All animals were treated under the rules and regulations held by the National Institutes of Health and Emory University Institutional Animal Care and Use Committee. The qk^v^ mouse colony was originally purchased from Jackson Laboratory and maintained on campus. The wt/qk^v^, qk^v^/qk^v^ and PLP-FLAG-QKI-6 transgenic mice were generated and identified by PCR-genotyping (23). After euthanization, brain stems, optic nerves (OPNs) and sciatic nerves (SCNs) were dissected, and processed for protein or RNA extraction.

### Plasmid constructs

The FLAG-tagged QKI-6 cDNA expression construct was previously described (13). hnRNP H 3′ untranslated region (3′UTR) reporter was made by PCR amplification of mouse genomic DNA using primers with engineered XbaI and XhoI restriction sites for cloning downstream of the firefly luciferase coding sequence. Mutagenesis to delete the entire bipartite sequence of the putative QKI-response element (QRE) in the hnRNP H reporter, and the G-runs in the MAG minigene (11) were performed using the QuikChange lightening mutagenesis kit (Agilent Technologies). All primers used for cloning and mutagenesis are listed in Supplementary Table S2.

### Cell cultures and transfections

The HOG, CG4 and CAD cell lines were propagated as previously described (18,29–30). Transfections were performed using Lipofectamine 2000 (Invitrogen) with DNA constructs and/or siRNAs following manufacturer's instructions. The FLAG-QKI-6 construct or pcDNA control plasmid (2 μg) was transfected into HOG cells on two sequential days, with 0.5 μg of reporter plasmids co-transfected on the first day. For the luciferase assay, all conditions were co-transfected with the Renilla plasmid. Firefly and Renilla luciferase activities were measured with a luminometer using dual luciferase assay reagents (Promega). Knockdown of hnRNP F/H was achieved by transfection with 100 pmol of a published siRNA (25) or a Silencer Negative Control #1 (Invitrogen) on two consecutive days. Same transfection conditions were performed for knockdown of hnRNP A1 using 50 pmol hnRNP A1 siRNA (Santa Cruz) (31). MAG minigene (1 μg) was co-transfected with siRNA on the second round of treatment. Cells were harvested 48 h after the first transfection for RNA or protein analysis.

### Protein/RNA preparation and quantification assays

Whole cell lysates were prepared by sonication in 1× Laemlli buffer (19) for sodium dodecyl sulphate-polyacrylamide gel electrophoresis (SDS-PAGE) immunoblot analysis with the following antibodies: anti-hnRNP F/H (1:4000; Abcam), anti-hnRNP A1 (1:5000; Sigma), anti-β-actin (1:10 000; Sigma), anti-Flag (1:2000; Sigma), anti-eIF5α (1:10 000; Santa Cruz). The pixel density of protein bands on X-ray films was quantified using Image J software.

RNA was isolated by Trizol extraction (Invitrogen). Samples were DNase treated with the Quantitect kit (Qiagen) prior to reverse transcription (RT) using random hexamer primers (Promega) and SSII reverse transcriptase (Invitrogen), or using the Quantitect kit. Semi-quantitative RT-PCR was performed using primers that detect the alternative splice variants simultaneously. After electrophoresis, the RT-PCR products of AS isoforms were quantified with ImageJ. The intensity of ethidium bromide staining of the PCR products that contain either exon included or skipped was quantified. The % inclusion of the alternatively spliced exon in each sample was calculated. Results in qk^v^/qk^v^ mutant or siRNA knockdown samples were statistically compared with the corresponding control in each experiment and graphically displayed. Real-time RT-PCR (qRT-PCR) was performed using DyNaMo Sybr Green qPCR kit (Thermo-Scientific), and quantified by ΔΔCt. Primers are listed in Supplementary Table S2, or were previously published (11,28,32–33).

### UV-crosslinking immunoprecipitation

Brain stems derived from mice expressing the FLAG-QKI-6 transgene specifically in OLs were dissected, minced and immediately UV cross-linked at 400 mJ in a Stratalinker 1800 (Agilent Technologies). In addition, HOG cells transfected with FLAG-QKI-6 or pcDNA control, together with reporter constructs, were also subjected to UV cross-link under the same conditions. Cells or homogenized tissues were lysed on ice in a buffer containing 20 mM Tris pH 7.5, 100 mM KCl, 5 mM MgCl2, 0.5% Triton X, a cocktail of protease inhibitors including pepstatin, leupeptin, aprotinin, phenylmethanesulfonyl fluoride and RNase inhibitor. Lysates were centrifuged at 800 × g for 5 min at 4°C to remove nuclei. SDS was added to supernatants to a final concentration of 0.01% or 0.5% as indicated in the corresponding figure legends and centrifuged at 16 000 × g for 15 min at 4°C. Four percent of total supernatant was kept for detecting RNA and protein inputs, respectively. Samples were pre-cleared for 30 min at 4°C with sepharose 4B beads (Sigma) that were pre-balanced in the IP buffer. Twenty microliters of pre-balanced anti-Flag-M2 beads (Sigma) were added to the supernatants and incubated overnight at 4°C. Centrifugation was performed at 100 × g at 4°C to isolate the immunoprecipitated complexes on the beads, which were washed three times in a buffer containing 20 mM Tris pH 7.5, 100 mM KCl, 5 mM MgCl2, protease inhibitors and 1% Triton X. The beads were then resuspended in a buffer containing 5 mM Tris pH 7.5, 0.32 M sucrose and 2% SDS. Seventy-five percent of the immunoprecipitates on beads were taken out and treated with proteinase K for 20 min. After centrifugation at 8000 × g, the RNA was isolated from the supernatant by phenol/chloroform extraction, and subjected to RT-PCR to detect FLAG-QKI-6 associated RNA. For experiments using reporter plasmids, samples were DNase treated before RT, with an independent replicate that was not reverse transcribed, to assess complete removal of plasmid DNA contamination. The remaining 25% of the immunoprecipitates were incubated at 65°C for 3 min in 1× Laemlli buffer, centrifuged, and the supernatant was subjected to SDS-PAGE and immunoblot.

### Statistical analysis

Statistical analyses were performed using Student's t-tests to compare two sample sets, one-way ANOVA and Tukey's post-test when comparing three or more sample sets, or two-way ANOVA and Bonferroni's post-test when comparing three or more sets of paired conditions. Standard errors are depicted in all bar graphs. *P*-value < 0.05 was considered statistically significant and is indicated by *.

## RESULTS

### QKI-6 deficiency in OLs is responsible for aberrant over-expression of hnRNP F/H proteins without affecting their mRNAs

**Figure 1.**
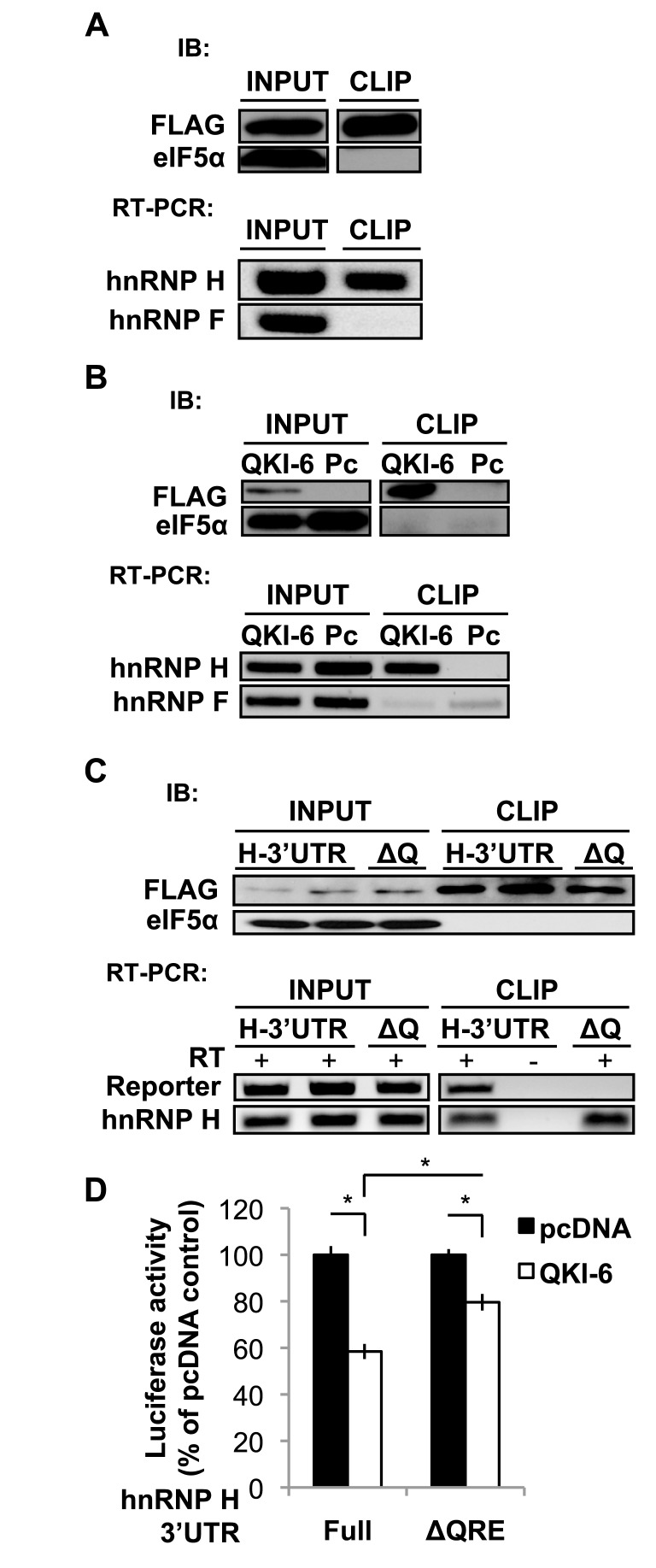
QKI-6 binds hnRNP H mRNA in a QRE-dependent manner, but not hnRNP F mRNA. (**A**) UV-crosslinking immunoprecipitation (CLIP) for co-isolation of mRNAs with FLAG-tagged QKI-6 expressed in OLs of the brain stem in the FLAG-QKI-6 transgenic mouse. Top panels: immunoblot detection of FLAG-QKI-6 in INPUT and CLIP complexes. The housekeeping protein elF5α is detected in INPUT but absent from CLIP. Bottom panels: RT-PCR detects hnRNP H mRNA by CLIP, but not hnRNP F mRNA. (**B**) CLIP of the human OL cell line HOG transfected with FLAG-QKI-6 plasmid (QKI-6) or pcDNA vector (Pc). Immunoblot indicates successful isolation of FLAG-QKI-6 (top panels) and RT-PCR indicates co-IP of hnRNP H mRNA, but not hnRNP F mRNA (bottom panels). (**C**) CLIP of HOG cells co-expressing FLAG-QKI-6 with a reporter either fused with mouse wild-type 3′UTR of hnRNP H (H-3′UTR) or a mutant 3′UTR that lacks the putative QRE (ΔQ). Similar amount of FLAG-QKI-6 is isolated from each lysate (top panel). The absence of the QRE diminished the ability for co-IP of the reporter mRNA with FLAG-QKI-6 by RT-PCR using primers specific for the reporter coding region, whereas endogenous hnRNP H mRNA still co-immunoprecipitated with FLAG-QKI-6 (bottom panel). Replicates from two independent transfections with the wild-type hnRNP H 3′UTR were subjected to CLIP in parallel, one of which was processed without reverse transcriptase (RT) as a negative control. All CLIP experiments were performed in the IP buffer containing 0.01% SDS. (**D**) Luciferase activity from hnRNP H reporters, wild-type (full) or mutant (ΔQRE) in HOG cells transfected by FLAG-QKI-6 construct or the pcDNA control vector. The firefly luciferase activity is normalized to the activity of co-expressed Renilla luciferase. Results are graphically displayed as percent of pcDNA control.

Our previous studies showed that QKI-6 is necessary for normal AS of the PLP pre-mRNA specifically expressed in OLs, which is a well-characterized target of hnRNP F/H (12,25,34). This raises a question whether QKI-6 may control AS through regulating hnRNP F and/or hnRNP H. Consistent with this idea, putative QREs are found in the 3′UTRs of hnRNP F and hnRNP H mRNAs, which are conserved in mouse, rat and human (Supplementary Figure S1). To test whether QKI-6 may interact with hnRNP F/H mRNAs in myelinating OLs *in vivo*, we performed UV-crosslinking immunoprecipitation (CLIP) to isolate mRNA complexes formed with cytoplasmic QKI-6 from the brain of transgenic mice that express FLAG-QKI-6 specifically in OLs (23). Successful isolation of FLAG-QKI-6 was indicated by immunoblot (Figure 1A, top panel) and the co-immunoprecipitated RNA was subjected to RT-PCR using hnRNP F- and hnRNP H-specific primer sets that target the 3′UTRs at or near the putative QREs. hnRNP H mRNA was clearly co-immunoprecipitated with FLAG-QKI-6 from OLs in the brain (Figure 1A, bottom panel). In contrast, despite abundant expression, hnRNP F mRNA was not co-isolated with FLAG-QKI-6. CLIP-RT-PCR also demonstrated that endogenous human hnRNP H mRNA, but not hnRNP F mRNA, was co-isolated with FLAG-QKI-6 expressed in the human OL cell line HOG (Figure 1B), which was attenuated without UV crosslinking (Supplementary Figure S2). In addition, fusing the 3′UTR of mouse hnRNP H to a reporter mRNA enabled an interaction with FLAG-QKI-6 in transfected HOG cells, which was impaired when the predicted bipartite QRE in the 3′UTR was entirely deleted (ΔQRE, Figure 1C). Moreover, when co-transfected into HOG cells, QKI-6 significantly suppressed expression of luciferase from the reporter that carries the hnRNP H 3′UTR, whereas deleting the putative QRE reduced the effect by QKI-6 (Figure 1D). Together, these data suggest that cytoplasmic QKI-6 selectively interacts with mouse and human hnRNP H mRNA, and negatively regulates hnRNP H in cells of the OL lineage by targeting the conserved QRE in the 3′UTR.

**Figure 2.**
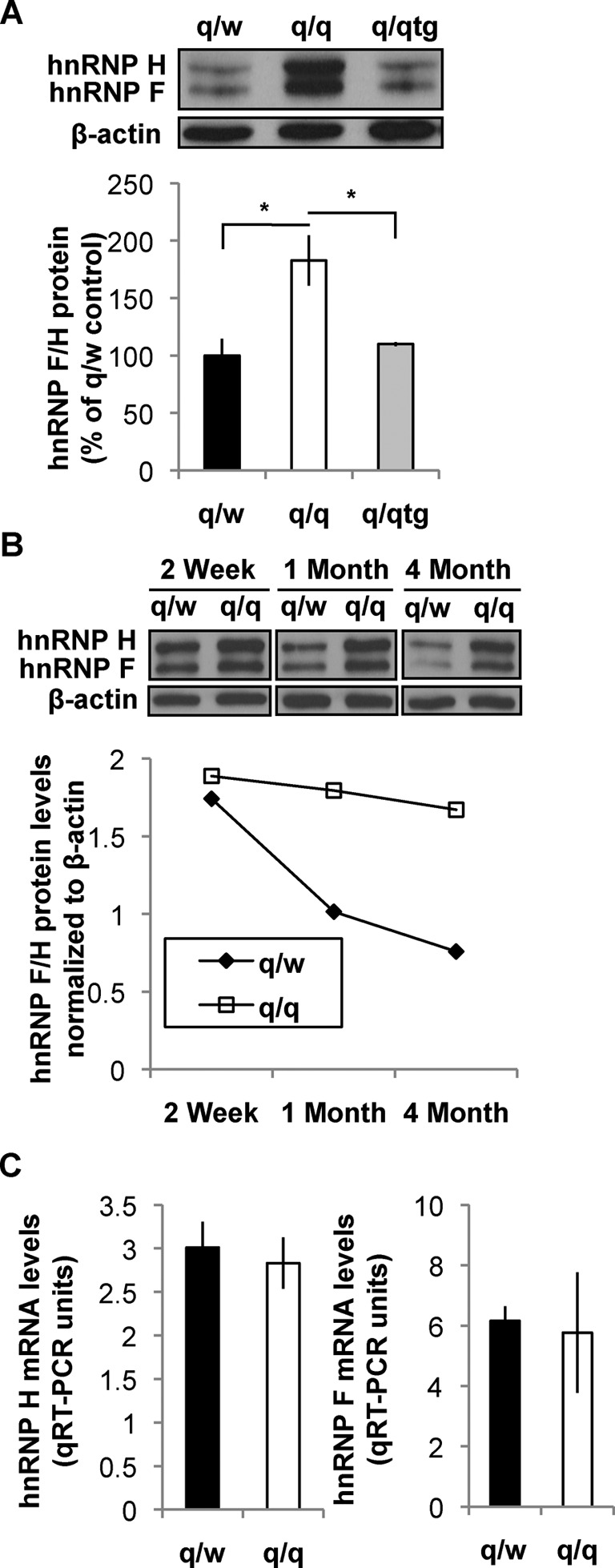
QKI-6 deficiency results in over-expression of hnRNP F/H in OLs during CNS myelin development without affecting the abundance of hnRNP F/H mRNAs. (**A**) Immunoblot of hnRNP F/H in adult (4–5 months) OPNs derived from the heterozygous qk^v^ (q/w) non-phenotypic control, homozygous qk^v^ mutant (q/q), and q/qtg mice that express FLAG-QKI-6 in OLs. β-actin was a loading control for quantification of hnRNP F/H. Percent changes of hnRNP F/H protein levels relative to that in q/w are graphically displayed in the bottom panel. (**B**) Immunoblot of hnRNP F/H in q/q and q/w OPNs during myelin development at the indicated postnatal age. Signal density of hnRNP F/H was normalized to β-actin and graphically displayed in the bottom panel. (**C**) qRT-PCR quantification of hnRNP H and F mRNAs isolated from adult OPNs of q/w and q/q mice normalized to β-actin mRNA readings by ΔΔCt.

We next examined whether and how QKI-6 regulates expression of hnRNP H during CNS myelin development. hnRNP H is broadly expressed in many cell types (1,24) whereas QKI deficiency in the CNS of the qk^v^/qk^v^ mutant mice is restricted to OLs (21). Therefore, we performed immunoblot analysis to quantify hnRNP H protein in the OPNs highly enriched of OLs. An antibody that detects both hnRNP F and H, which display distinct sizes on SDS-PAGE, was used. Surprisingly, both hnRNP F and H were aberrantly increased approximately 2-fold in the qk^v^/qk^v^ OPNs as compared to that in the non-phenotypic heterozygous qk^v^/wt control (Figure 2A), despite the lack of interaction between QKI-6 and the hnRNP F mRNA (Figure 1). Moreover, transgenic expression of FLAG-QKI-6 alone specifically in qk^v^/qk^v^ OLs completely reversed hnRNP F/H proteins to normal levels. These results clearly indicate that both hnRNP F and hnRNP H are functional targets of QKI-6 in OLs. We further showed that both hnRNP F and H were down-regulated in the OPNs during normal myelin development whereas QKI deficiency in the qk^v^/qk^v^ OLs impaired such regulation (Figure 2B). However, despite the significantly elevated expression of hnRNP F/H proteins in qk^v^/qk^v^ OPNs, the steady-state levels of the hnRNP F/H mRNAs remain normal (Figure 2C). In addition, a detectable increase in polyribosome association was observed for both the hnRNP H and hnRNP F mRNAs in the brain stem of qk^v^/qk^v^ as compared to that in the qk^v^/wt littermate control based on linear sucrose gradient fractionation followed by qRT-PCR (data not shown). Consistent with the known function of QKI-6 in suppressing translation of its bound mRNAs (11,20), these results suggest that QKI-6 may directly repress translation of its bound hnRNP H mRNA and indirectly repress translation of the hnRNP F mRNA through yet undefined mechanisms.

### hnRNP F/H targets G-run elements to regulate inclusion of the alternative exon in the MAG pre-mRNA, which is dysregulated in the qk^v^/qk^v^ mutant

**Figure 3.**
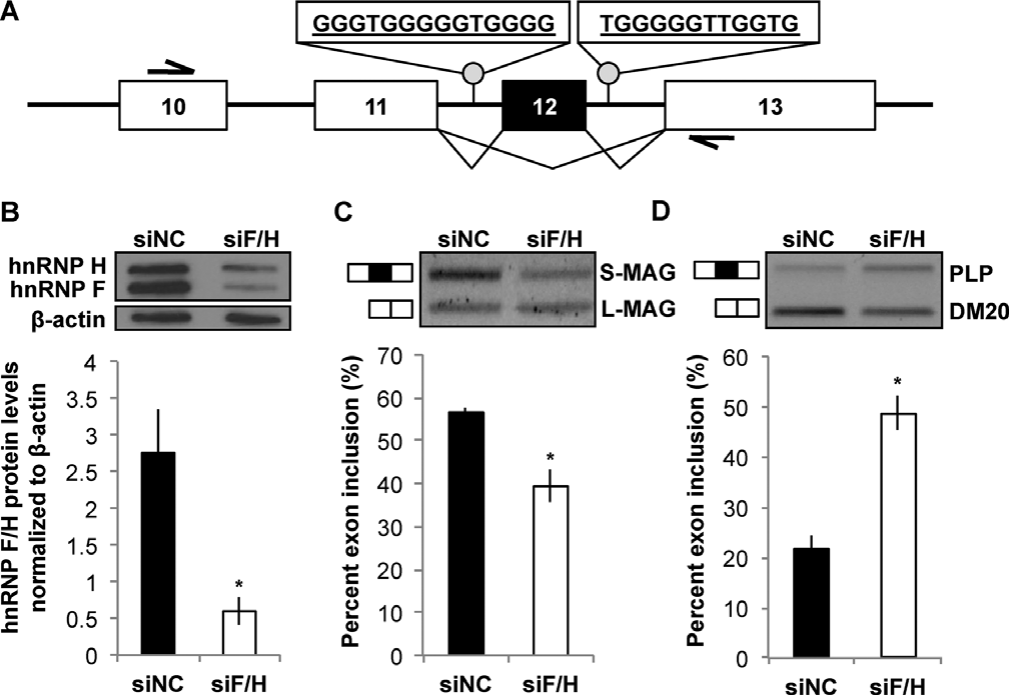
hnRNP F/H promotes inclusion of Exon 12 in MAG pre-mRNA. (**A**) Schematic of inclusion or exclusion of the alternative Exon 12 (marked black) in MAG pre-mRNA. Exons are boxed/numbered, introns are displayed as lines, and intronic G-run (rat) sequences are indicated. Primers that simultaneously detect AS isoforms of MAG mRNAs are depicted by half-arrows. (**B**) Immunoblot detects siRNA knockdown of hnRNP F/H (siF/H) as compared to negative control siRNA (siNC) in CG4 cells (top panel). Signal density of hnRNP F/H was normalized to β-actin and graphically displayed (bottom panel). (**C** and **D**) Representative image of semi-quantitative RT-PCR products (top panels) of alternatively spliced endogenous MAG pre-mRNA (C, S-MAG and L-MAG) and PLP pre-mRNA (D, PLP/DM20). The alternative exon is marked black and inclusion is depicted on the side for the corresponding band. The % inclusion of the alternative exon in each sample is calculated and results are statistically compared between siF/H and siNC-treated cells (bottom panels).

We next asked whether hnRNP F/H might control AS of pre-mRNAs that are dysregulated in the qk^v^/qk^v^ OLs. Inclusion of MAG Exon 12 is aberrantly increased in qk^v^/qk^v^ OLs (11), which is rescued by the FLAG-QKI-6 transgene (11,23). Noticeably, long stretches of G-runs, analogous to the consensus sequence targeted by hnRNP F/H in numerous pre-mRNAs (25,34–35), are found in the introns within 250 nt of both the 3′ and 5′ splice sites that define Exon 12 in the mouse, rat and human MAG gene (Supplementary Figure S3), all within optimal distance known for regulation by hnRNP F/H (26). To test whether hnRNP F/H may regulate AS of endogenous MAG pre-mRNA that harbor G-runs flanking Exon 12 (Figure 3A), we knocked down hnRNP F and hnRNP H simultaneously in the rat OL cell line CG4 (Figure 3B) using a previously validated siRNA (25). As a result, inclusion of Exon 12 was significantly reduced based on semi-quantitative RT-PCR that detects both MAG mRNA isoforms with a single primer set (Figure 3C). Knockdown hnRNP F/H in CG4 cells in a parallel experiment also significantly affected AS of the PLP pre-mRNA (Figure 3D), which is a well-characterized target of hnRNP F/H (25,28,34).

**Figure 4.**
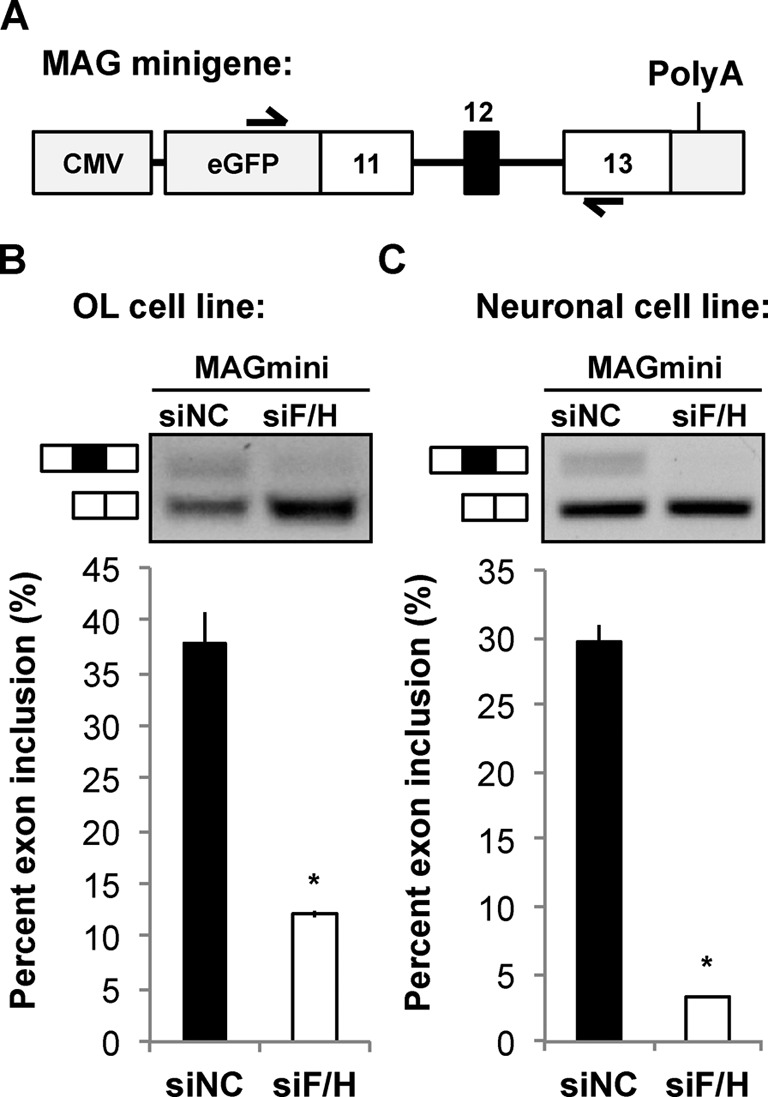
hnRNP F/H regulates AS of a MAG minigene in OL and neuronal cell lines. (**A**) Schematic of the MAG minigene reporter construct in pEGFPC2 plasmid. Half-arrows indicate forward and reverse primers for simultaneous detection of AS isoform mRNAs from the MAG minigene. (**B** and **C**) Representative image of semi-quantitative RT-PCR products (top panels) upon treatment of the OL cell line CG4 (B) and the neuronal cell line CAD (C) with an siRNA to knockdown hnRNP F/H or a negative control siRNA (siNC). The % inclusion of the alternative exon in each sample is calculated and results are statistically compared between siF/H and siNC-treated cells (bottom panels).

**Figure 5.**
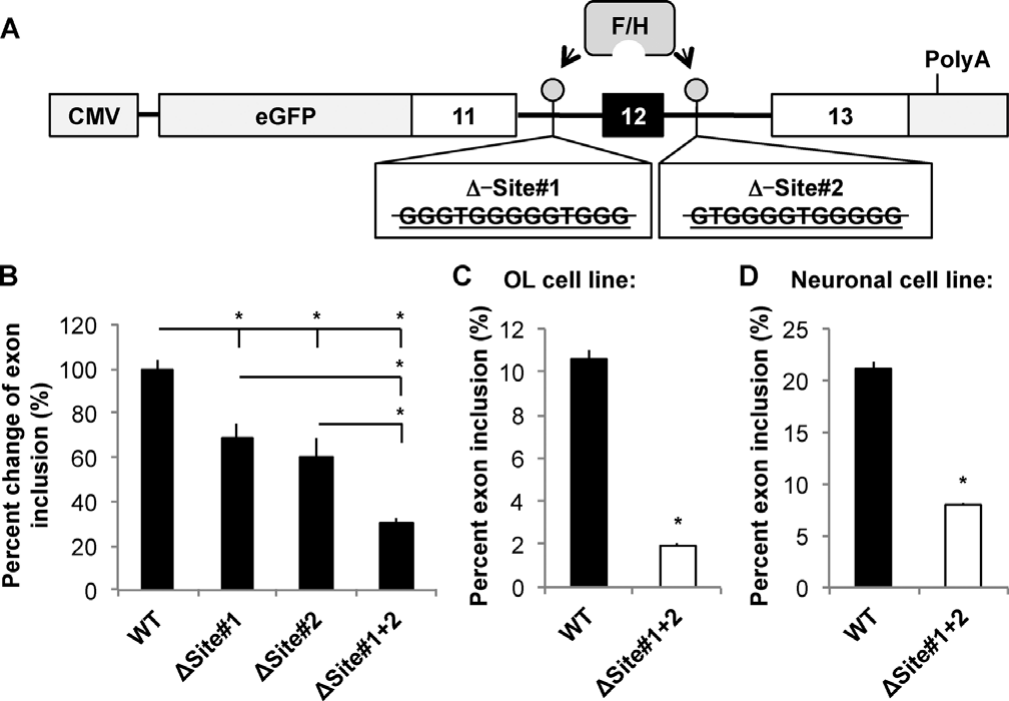
Inclusion of MAG Exon 12 is reduced upon deletion of intronic G-runs. (**A**) Schematic of the MAG minigene reporter constructs illustrating deletion of individual or both intronic G-runs (mouse sequence). (**B**) qRT-PCR detection of mRNA isoforms derived from AS of the MAG minigene reporter that carries wild-type intronic G-runs (WT), deletion of the 5′-G-run (ΔSite#1), 3′-G-run (ΔSite#2) or both G-runs (ΔSite#1+2) expressed in the neuronal cell line CAD. Minigene-isoform-specific primers (11) were used for qPCR, and the ΔΔCt of inclusion versus exclusion was calculated. The % change of Exon 12 inclusion in each mutant minigene was calculated and statistically compared with the WT minigene. (**C** and **D**) Semi-quantitative RT-PCR detects reduced Exon 12 inclusion of the MAG minigene when both G-runs are deleted (ΔSite#1+2) in the OL cell line CG4 (C) and the neuronal cell line CAD (D). The same primer set used in Figure 4 was employed for (C) and (D). The % inclusion of the alternative exon in each sample is calculated and results are statistically compared between the WT and mutant minigene.

To directly test whether hnRNP F/H target the G-run elements to control inclusion of MAG Exon 12, we utilized a MAG minigene in which the genomic sequence for regulating AS of Exon 12 was fused in-frame downstream of the EGFP coding sequence (Figure 4A). Upon knockdown of hnRNP F/H, Exon 12 inclusion from the MAG minigene was significantly reduced in transfected CG4 cells (Figure 4B). Interestingly, hnRNP F/H knockdown in the neuronal cell line CAD that does not naturally express MAG also reduced Exon 12 inclusion from the minigene (Figure 4C). This result suggests that hnRNP F/H can regulate inclusion of MAG Exon 12 independent of OL-specific factors. Importantly, deletion of either G-run alone from the minigene, as illustrated in Figure 5A, reduced Exon 12 inclusion by 30–40% based on qRT-PCR (Figure 5B). Moreover, deletion of both G-runs led to an additive effect, resulting in greater than 60% reduction of Exon 12 inclusion (Figure 5B), which largely recapitulates the effects caused by hnRNP F/H knockdown (Figure 4B). Such effects were observed in CG4 and CAD cells (Figure 5C and D. These results identify both G-runs as equivalent functional targets of hnRNP F/H for regulating MAG Exon 12 inclusion. Furthermore, although hnRNP A1 also regulates AS of MAG (11), the G-run deletion still causes comparable reduction of Exon 12 inclusion when hnRNP A1 is knocked down (Supplementary Figure S4). This result suggests that hnRNP F/H can regulate inclusion of MAG Exon 12 through the G-runs independent of hnRNP A1.

### QKI deficiency affects the hnRNP F/H-dependent AS pathway in myelinating glia of the CNS and PNS

**Figure 6.**
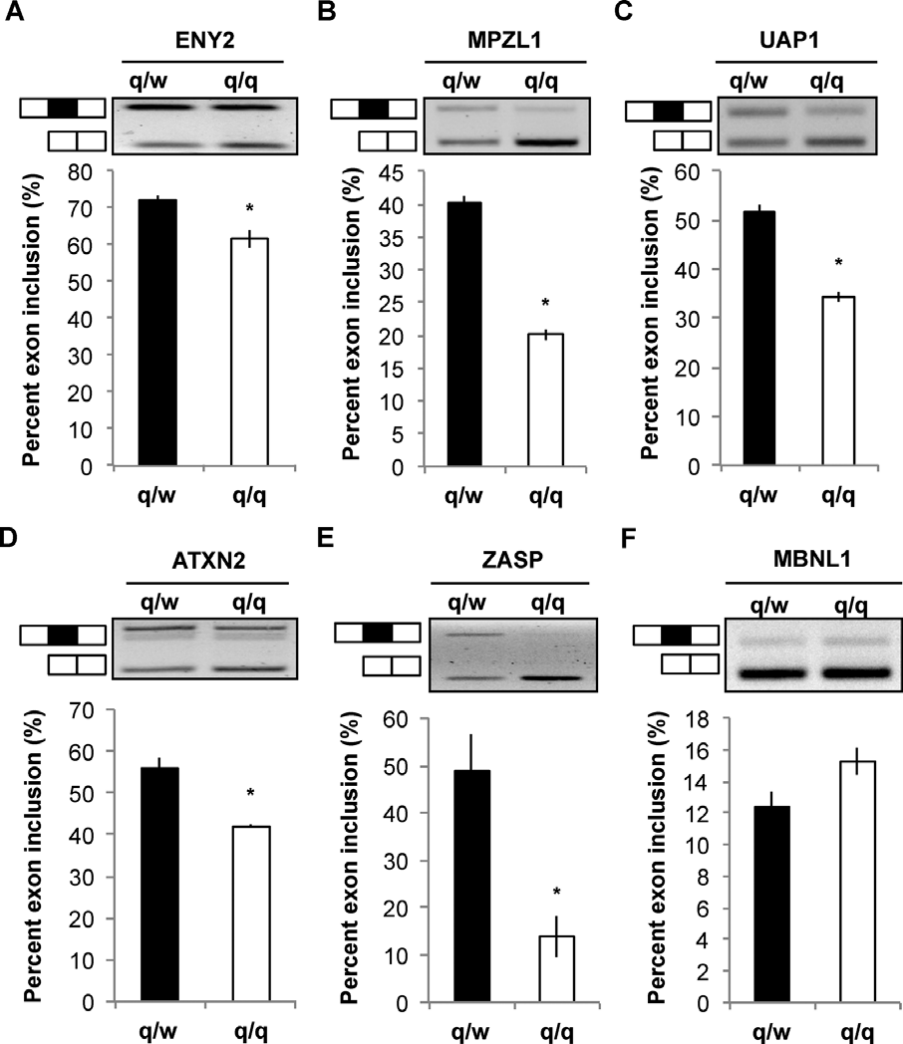
Alternative splicing of hnRNP F/H target mRNAs are dysregulated in the qk^v^/qk^v^ mouse. Semi-quantitative RT-PCR using primers that simultaneously detect AS isoforms of ENY2 (**A**), MPZL1 (**B**), UAP1 (**C**), ATXN2 (**D**), ZASP (**E**) and MBNL1 (**F**) in the OPNs of q/w and q/q mice. Representative images of the semi-quantitative RT-PCR products derived from the splice variants of the aforementioned genes are shown in the top panels. The alternative exons in the PCR products are indicated in black and inclusion is depicted on the side of the corresponding bands. The % inclusion of the alternative exon in each sample is calculated and results are statistically compared between the q/q mutant and the q/w control in the bottom panels.

To test whether QKI regulates AS of additional pre-mRNAs targeted by hnRNP F/H, we identified and examined 26 putative functional targets of hnRNP F/H, including PLP and MAG. These pre-mRNAs either contain an alternative exon flanked by G-runs or display altered AS in response to changes of hnRNP F/H expression in various cell types, including a mouse OPC line Oli-neu (27–28,33,36–37) (Supplementary Table S1). A number of these pre-mRNAs are also known to bind human hnRNP H in HEK293T cells through G-run motifs, as revealed by CLIP-RNA sequencing (CLIP-seq) (26,38). We detected mRNA expression from all the aforementioned genes in OLs or OPNs by RT-PCR. Among these transcripts, 19 generate alternative mRNA splice variants, and are potential hnRNP F/H targets in OLs. In addition, we observed dysregulated AS in eight of these hnRNP F/H targets in the qk^v^/qk^v^ OPNs (Supplementary Table S1). Quantification of exon inclusion/exclusion is shown in Figure 6. Among these dysregulated AS targets, the enhancer of yellow 2 transcription factor homolog (ENY2) was shown to be regulated by hnRNP F/H in OPCs in a recent report (28). Consistent with the finding that siRNA-mediated knockdown of hnRNP F/H in OPCs enhanced inclusion of the alternative exon in ENY2 (28), the abnormal over-expression of hnRNP F/H in the qk^v^/qk^v^ OPNs repressed exon inclusion of ENY2 (Figure 6A). In addition, dysregulated AS in qk^v^/qk^v^ OPNs was also observed in the pre-mRNAs encoding myelin protein zero-like 1 (MPZL1), UDP-acetylglucosamine pyrophosphorylase 1 (UAP1), ataxin-2 (ATXN2), and Z-band alternatively spliced PDZ-motif containing protein (ZASP) (Figure 6B–E). Hence, QKI regulates hnRNP F/H-dependent AS to control expression of genes that play important roles in CNS myelination, metabolism, synaptic function and neuronal degeneration (11,25,39–40).

Not all the reported hnRNP F/H targets are dysregulated by QKI deficiency, represented by the muscle blind-like protein 1 (MBNL1) (Figure 6F). Consistent with the previously reported regulation of AS of MBNL1 by hnRNP F/H in skeletal muscle cells (33), siRNA knockdown of hnRNP F/H significantly altered AS of MBNL1 in the CAD neuronal cell line (Supplementary Figure S5A). However, AS of MBNL1 was not affected in the OL cell line CG4 upon knockdown of hnRNP F/H (Supplementary Figure S5B). This result suggests that the functional influence of hnRNP F/H on AS of MBNL1 might be masked by OL-specific factors. In contrast, AS of ATXN2 was equivalently affected by hnRNP F/H knockdown in both the CAD and CG4 cell lines (Supplementary Figure S5C and D), suggesting that hnRNP F/H regulates AS of ATXN2 in both neurons and OLs.

**Figure 7.**
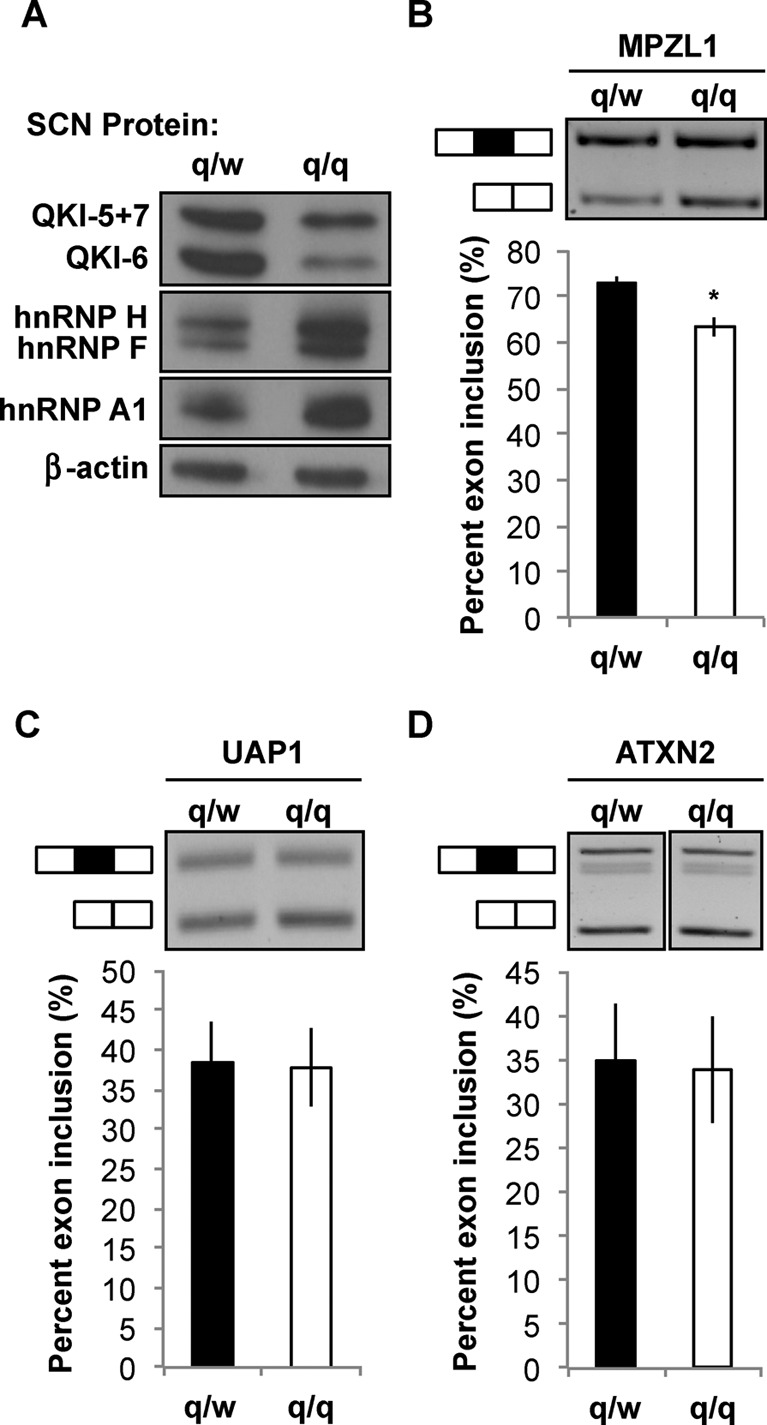
The hnRNP F/H targets are differentially affected in Schwann cells as compared to OLs by QKI deficiency. (**A**) Immunoblot detects QKI deficiency and aberrant over-expression of hnRNP F, H and A1 in the Schwann-cell-rich sciatic nerve (SCN) of the homozygous qk^v^ mice (q/q) as compared to the heterozygous non-phenotypic control (q/w). β-actin serves as the loading control. (**B**–**D**) Semi-quantitative RT-PCR of RNA isolated from q/w and q/q SCNs for quantification of MPZL1 (B), UAP1 (C) and ATXN2 (D) as described in the legend of Figure 6. Representative RT-PCR products of the splice variants are shown in the top panels. The % inclusion of the alternative exon in each sample is calculated and results are statistically compared between the q/q mutant and the q/w control in the bottom panels.

The qk^v^ mutation also causes QKI deficiency in SWCs that myelinate the PNS (14). QKI-6 is preferentially reduced among QKI isoforms in the qk^v^/qk^v^ SCN (Figure 7A), which is highly enriched of SWCs (41). Both hnRNP F and hnRNP H proteins were abnormally up-regulated in the qk^v^/qk^v^ SCNs, recapitulating what was found in qk^v^/qk^v^ OPNs. Elevated expression of hnRNP A1, a known target of QKI-6 in OLs (11), was also detected in the qk^v^/qk^v^ SCNs (Figure 7A). Thus, the functional consequence of QKI-6 deficiency on splicing factor expression appears to be comparable in both the CNS and the PNS. Among our identified hnRNP F/H targets that display abnormal AS in the qk^v^/qk^v^ OPNs, the MPZL1 pre-mRNA also displayed dysregulated AS in the qk^v^/qk^v^ SCNs (Figure 7B). However, unlike the dysregulated AS of UAP1 and ATXN2 in qk^v^/qk^v^ OPNs (Figure 6), transcripts from neither gene were affected in the qk^v^/qk^v^ SCNs (Figure 7C and D. Thus, QKI deficiency differentially affects AS of hnRNP F/H targets in myelinating glia from the CNS and PNS, presumably due to modulation of hnRNP F/H-dependent AS by undefined SWC-specific factors.

## DISCUSSION

Our studies discovered that QKI acts upstream of two canonical splicing factors hnRNP F/H, which forms a novel post-transcriptional pathway to regulate AS in myelinating glia of the CNS and PNS. We found that in contrast to nuclear QKI-5 that directly regulates AS in muscle cells (22), cytoplasmic QKI-6 controls AS in myelinating glia by repressing expression of hnRNP F/H. To our knowledge, QKI-6 is the first factor identified that governs the cellular abundance of hnRNP F/H and selective exon inclusion of hnRNP F/H targets. Furthermore, we identified *in vivo* functional targets of the QKI-6-hnRNP F/H pathway, which play diverse roles in glia function and myelin development, some of which are also implicated in neurological diseases.

hnRNP F and hnRNP H are functional orthologues expressed in many cell types and control AS of numerous G-run containing pre-mRNAs (1,24–25). CLIP-Seq has identified hundreds of human pre-mRNAs bound by hnRNP H through G-run motifs in HEK293T cells (26,38). In addition, AS of a number of pre-mRNAs are regulated upon manipulation of hnRNP F/H expression in other human cell types (27,33,36–37). Furthermore, a recent report discovered 190 pre-mRNAs that display altered splicing in response to siRNA knockdown of hnRNP F/H in a mouse OPC line (28). However, there is minimal overlap between these published human and mouse hnRNP F/H targets. Nonetheless, all of the functional AS targets of hnRNP F/H we identified in OLs (Supplementary Table S1) contain G-runs flanking the alternatively spliced exons in both the human and the mouse gene. The locations of these G-runs are within optimal distance for regulating AS based on large number of CLIP-seq targets of hnRNP H (26,28,38). In fact, a number of the transcripts are also CLIP-seq targets of hnRNP H, represented by ATXN2 (38). The functional importance of conserved G-runs in MAG and PLP is validated by mutation studies (Figure 5) (25). In addition, a conserved QRE is present in the human and the rodent hnRNP H 3′UTR. Thus, AS under control of the QKI-6-hnRNP F/H pathway appears to be conserved between mouse and human. One possible explanation for the diversity in previously reported mouse and human hnRNP F/H targets could be the existence of cell type-specific factors that modulate hnRNP F/H-dependent AS.

Regulation of hnRNP F/H expression by cytoplasmic QKI-6 provides one example for modulating hnRNP F/H-dependent AS in a cell type-specific manner. Expression of the qkI gene is detected in nearly all cell types except brain neurons (21). Nuclear QKI-5 is the predominant isoform in most cell types examined, which can directly regulate AS of its bound pre-mRNAs (21,22). In contrast, cytoplasmic QKI-6 is the most abundant QKI isoform in myelinating glia (21,23). Moreover, the cytoplasmic isoforms QKI-6 and QKI-7 are vigorously up-regulated during CNS myelin development, whereas QKI-5 and hnRNP F/H are down-regulated (8,14,25,28). In the qk^v^/qk^v^ OLs, although all QKI isoforms are reduced, deficiency of QKI-6 quantitatively exceeds the other isoforms (23). The impairment of hnRNP F/H decline in the qk^v^/qk^v^ OLs during myelin development, together with the correction of aberrant hnRNP F/H over-expression by the OL-specific FLAG-QKI-6 transgene, clearly demonstrates the essential role of QKI-6 in regulating hnRNP F/H and their downstream AS targets in CNS myelination. Importantly, the FLAG-QKI-6 transgene neither rescued the deficiency of QKI-5 (23) nor increased the nuclear abundance of QKI-5 (11). Hence, although QKI-5 regulates AS in other cell types (10,22,42), the deficiency of QKI-6 appears to be the major mechanism that affects hnRNP F/H-dependent splicing in myelinating glia.

Although QKI isoforms can form heterodimers and shuttle between the nucleus and the cytoplasm (16), both the endogenous QKI-6 and the FLAG-QKI-6 transgene product are predominantly localized to the cytoplasm of mature OLs *in vivo* (11,14,16,23). Consistent with the known function of QKI-6 in repressing mRNA translation (11), QKI deficiency in qk^v^/qk^v^ OLs leads to over-expression of hnRNP F/H proteins without affecting the levels of their mRNAs. However, we were unable to detect association of QKI-6 with the hnRNP F mRNA in OLs even under non-denaturing conditions. This could possibly be caused by unknown RNA-binding proteins that mask the QRE in hnRNP F mRNA, which either renders the hnRNP F mRNA inaccessible by QKI-6 or permits only labile interactions with QKI-6 in OLs. Considering the emerging evidence that QKI regulates expression of selected microRNAs (42,43), whether QKI-6-mediated miRNA expression also inhibits hnRNP F translation is an intriguing possibility to be explored by future studies.

All the AS targets of the QKI-6-hnRNP F/H pathway we identified in this study encode protein isoforms that play distinct functions, often reciprocally regulated during myelin development. MAG and PLP are two examples of such regulation, which are also severely dysregulated in the qk^v^/qk^v^ mutant (10–12). AS of MAG generates two transmembrane proteins that differ in their cytoplasmic domains, and hence are involved in distinct cellular signaling pathways that promote myelin formation (11,44). AS of PLP also generates two protein isoforms that perform non-overlapping functions in myelin-axon integrity (12,45). It is important to note that an AS event could be regulated by the cooperation of multiple splicing factors. In particular, QKI-6 also represses translation of hnRNP A1 (11,46), which also affects AS of MAG pre-mRNA *in vivo* in the same direction as that by hnRNP F/H. However, besides coordination with hnRNP H (47), hnRNPA1 was also shown to oppose hnRNP F in regulating AS of the insulin receptor pre-mRNA (48). The hnRNP F/H targets we analyzed could potentially be regulated by the coordination of multiple splicing factors downstream of QKI specifically in myelinating glia. Whether QKI-6 regulates additional splicing factors and how such regulation may cooperate to control AS in different cell types are challenging questions to be addressed by future studies.

Emerging evidence suggests dysregulation of AS in human disorders that involve deficiency of QKI, such as schizophrenia and glioma (8,49). Reduction of QKI isoforms and myelin-specific targets of QKI are observed in schizophrenic brains, including MAG and PLP (50). However, whether hnRNP F/H and/or hnRNP A1 are dysregulated, and furthermore to what extent the downstream AS pathway is affected, which in turn contribute to the myelin impairments in schizophrenia, still remain elusive. On the other hand, aberrant over-expression of hnRNP F/H and dysregulated AS of hnRNP F/H targets were found in human glioma specimens (27). In particular, hnRNP H was found to control an oncogenic splicing switch in gliomas, and was reported to promote expression of proliferation-inducing genes (27,28). Consistent with the repressive role of QKI in hnRNP F/H expression, the human qkI locus is frequently mutated in glioblastoma multiforme (GBM) derived from astrocytic origin identified by SNP-Chip analysis (51). Hence, besides its role in myelinating glia, QKI-6 may regulate hnRNP F/H-dependent AS to control normal growth and function of astrocytic glia. Whether the aberrant elevation of these hnRNPs in GBMs is caused by QKI-6 deficiency remains to be determined. Moreover, whether QKI-6 acts as a tumor suppressor to inhibit GBM tumorigenesis is an important question that warrants rigorous investigation.

## SUPPLEMENTARY DATA

Supplementary Data are available at NAR Online.

SUPPLEMENTARY DATA
