# Supplementary material for: A cytoplasmic quaking I isoform regulates the hnRNP F/H-dependent alternative splicing pathway in myelinating glia
Source: Nucleic Acids Res. 2014 May 3;42(11):7319–29. doi: 10.1093/nar/gku353 (PMC4066780; doi:10.1093/nar/gku353)
Supplement: SUPPLEMENTARY DATA [file supp_42_11_7319__index.html]

A cytoplasmic quaking I isoform regulates the hnRNP F/H-dependent alternative splicing pathway in myelinating glia — SUPPLEMENTARY DATA 

# A cytoplasmic quaking I isoform regulates the hnRNP F/H-dependent alternative splicing pathway in myelinating glia

## SUPPLEMENTARY DATA

**Files in this Data Supplement:**

- SUPPLEMENTARY DATA
