# Supplementary material for: A cytoplasmic quaking I isoform regulates the hnRNP F/H-dependent alternative splicing pathway in myelinating glia
Source: Nucleic Acids Res. 2014 May 3;42(11):7319–29. doi: 10.1093/nar/gku353 (PMC4066780; doi:10.1093/nar/gku353)
Supplement: SUPPLEMENTARY DATA [file supp_gku353_Supplementary_Data.pdf]

Supplementary Figure 1.

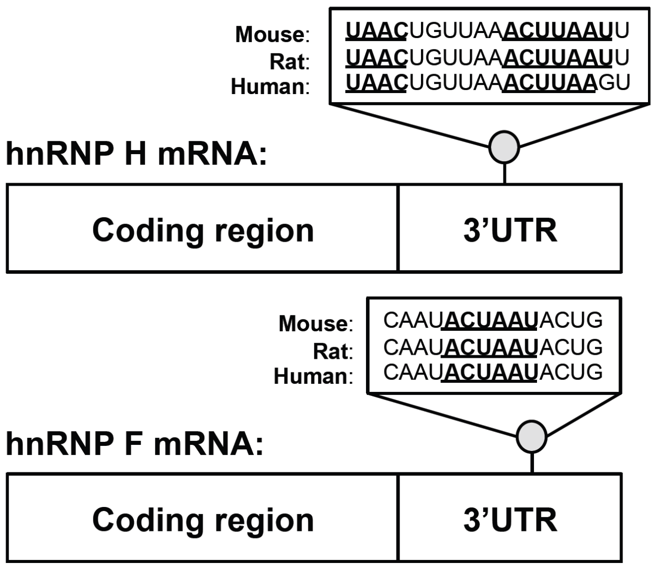

Putative quaking response elements (QRE) are conserved between rodents and human. The putative QREs are underlined in the 3' untranslated region (3'UTR) of mouse, rat and human hnRNP H and hnRNP F mRNAs.

Supplementary Figure 2.

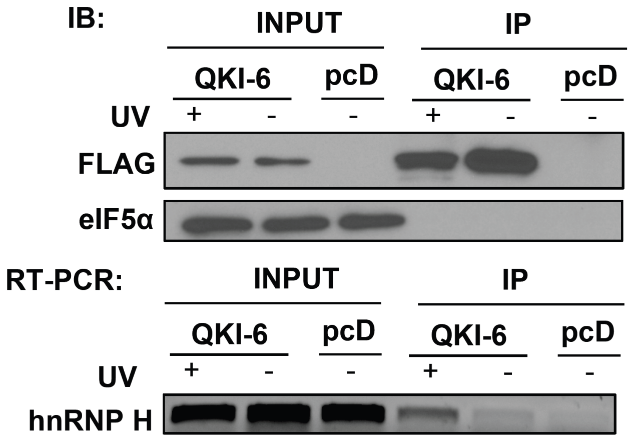

Co-immunoprecipitation with QKI-6 by the hnRNP H mRNA under denaturing conditions is attenuated without UV crosslinking. Immunoprecipitation from HOG cells transfected with FLAG-QKI-6 or pcDNA control was performed in the presence of 0.5% SDS, with or without UV crosslinking as indicated on top of the corresponding lanes. Before immunoprecipitation, cell lysates were subjected to ultracentrifugation at 77,000 xg for 20 min at 4°C. Immunoblots of IPs were probed with anti-FLAG antibody to detect FLAG-QKI-6 and anti-eIF5α antibody for loading control (top). hnRNP H mRNA in the input and IP was detected by RT-PCR (bottom).

Supplementary Figure 3.

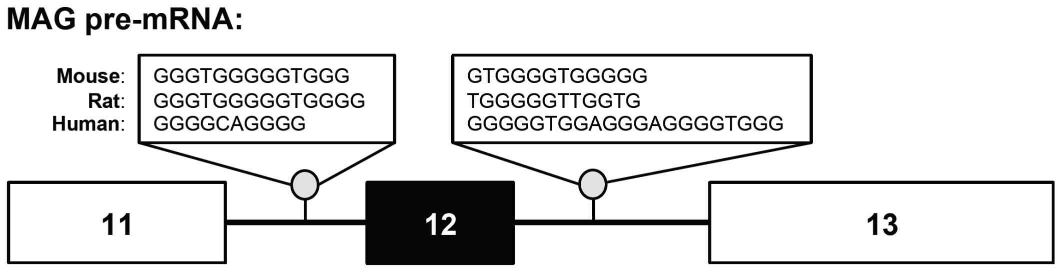

G-run motifs are identified surrounding MAG Exon 12 in rodents and human. Intronic G-runs are present within 250 base pairs upstream and downstream of the alternative Exon 12 in human, mouse, and rat MAG pre-mRNA.

Supplementary Figure 4.

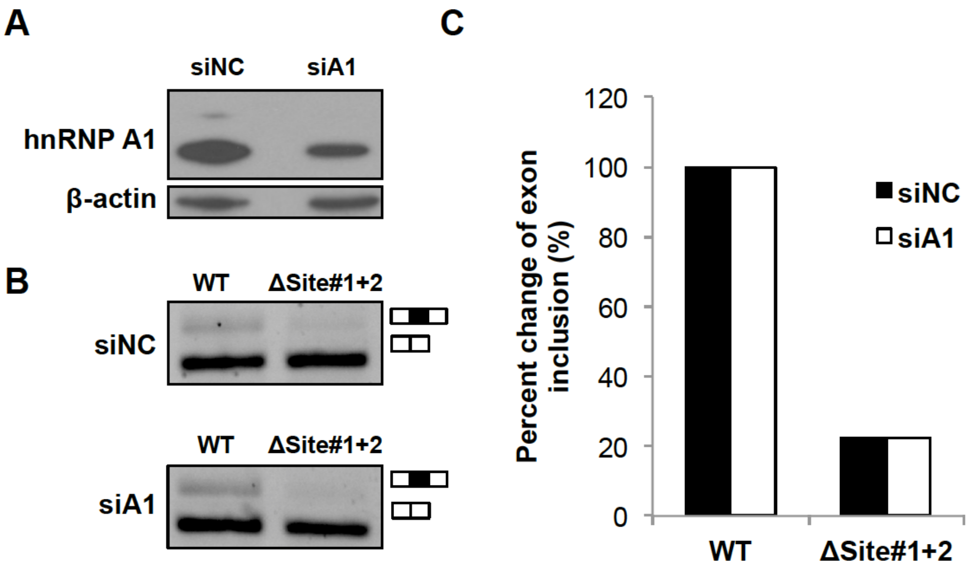

Deletion of G-runs in the MAG minigene results in comparable reduction of Exon 12 inclusion regardless the knockdown of hnRNP A1. (**A**) Immunoblot detects siRNA knockdown of hnRNP A1 (siA1) as compared to negative control siRNA (siNC) in CAD cells. β-actin was used as a loading control. (**B**) Representative image of semi-quantitative RT-PCR products of mRNA isoforms derived from AS of the MAG minigene reporter that carries wild type intronic G-runs (WT), or deletion of both G-runs (ΔSite#1+2), upon treatment with siNC or siA1. (**C**) qRT-PCR detection of mRNA isoforms derived from AS of the WT or mutant MAG minigene reporter in response to siNC or siA1. % change of inclusion of the alternative exon is calculated and graphically displayed (n=2).

Supplementary Figure 5.

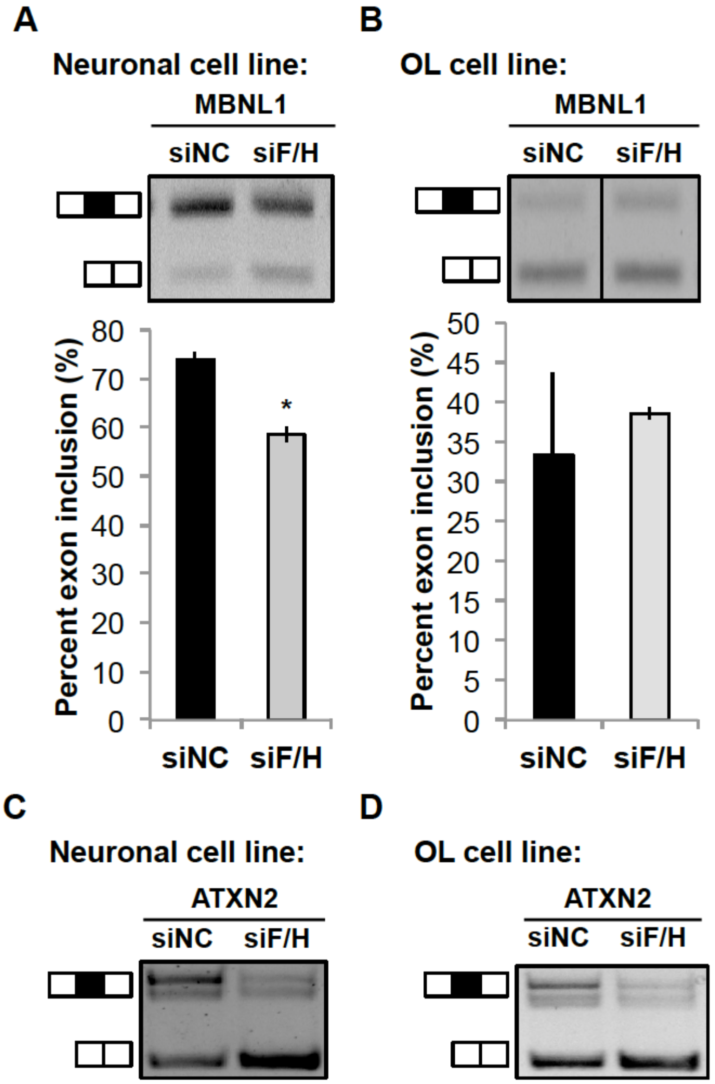

hnRNP F/H-dependent alternative splicing of MBNL1 and ATXN2 in neuronal and OL cell lines. (**A**) Semi-quantitative RT-PCR was performed using one primer set to simultaneously detect splice variants of MBNL1 in the neuronal cell line CAD upon siRNA knockdown of hnRNP F/H (siF/H) as compared to scrambled negative control siRNA (siNC). (**B**) Same experiment was performed in the OL cell line CG4. Representative images are shown in the top panels. The alternative exons in the PCR products are indicated in black and inclusion is depicted on the side of the corresponding bands. % inclusion of the alternative exon in each experiment is calculated and statistically compared between siF/H and siNC-treated cells in the bottom panels. (**C,D**) Representative image of semi-quantitative RT-PCR of ATXN2 upon knockdown of hnRNP F/H compared to scrambled negative control in the neuronal cell line CAD and the OL cell line CG4.

Supplementary Table 1.

Gene name	Gene Id (mouse)	mRNA in OLs	Splice variants in OLs	Abnormal AS in qk^v/qk^v	hnRNP H CLIP-tag in HEK293T	Species/Cell-types examined from literature	References
PLP	18823	Yes	Yes	Yes		Mouse, Oli-neu	(25)
MAG	17136	Yes	Yes	Yes		Mouse	This study
ENY2	223527	Yes	Yes	Yes		Mouse, Oli-neu	(28)
ZASP/LDB3	24131	Yes	Yes	Yes		Human, Skmc	(33)
UAP1	107652	Yes	Yes	Yes		Human, HeLa	(37)
MPZL1	68481	Yes	Yes	Yes		Human, HeLa	(37)
ATXN2	20239	Yes	Yes	Yes	✓	Human, HEK293T	(37)
NF2	18016	Yes	Yes	Yes		Human, HeLa	(38)
SIRT2	64383	Yes	Yes	No	✓	Human, HEK293T	(38)
MBNL1	56758	Yes	Yes	No		Human, Skmc	(33)
MBNL2	105559	Yes	Yes	No		Human, Skmc; mouse Oli-neu	(33) (37)
SMC2	14211	Yes	Yes	No		Mouse, Oli-neu	(28)
ELP3	74195	Yes	Yes	No		Mouse, Oli-neu	(28)
NFIB	18028	Yes	Yes	No		Mouse, Oli-neu	(28)
MADD	228355	Yes	Yes	No	✓	Human, HEK293T, HeLa, U373	(27) (38)
PTB	19205	Yes	Yes	No		Mouse	This study
nPTB	56195	Yes	Yes	No		Mouse	This study
FGFR2	14182	Yes	No	No		Human, HEK293T	(36)
TPM1	22003	Yes	No	No		Human, HeLa	(37)
BAT2 Domain	226562	Yes	Yes	N/A		Mouse, Oli-neu	(28)
DDEF1	13196	Yes	Yes	N/A		Mouse, Oli-neu	(28)
DSCR3 (CG4)	13185	Yes	No	N/A	✓	Human, HEK293T	(38)
HGS (CG4)	15239	Yes	No	N/A	✓	Human, HEK293T	(38)
TARBP2 (CG4)	21357	Yes	No	N/A	✓	Human, HEK293T	(38)
TRMU (CG4)	72026	Yes	No	N/A	✓	Human, HEK293T	(38)
MAST1 (CG4)	56527	Yes	No	N/A	✓	Human, HEK293T	(38)

Supplementary Table 2.

Primer List	
Name of Target	Sequence
Actin	F:TGTTACCAACTGGGACGACA
	R:GGGGTGTTGAAGGTCTCAAA
ATXN2	F:GTTCAGCACCATCAGCACCAG
	R:GGAGGGGATTTGGCCTTTCG
DSCR3	F:CGTGCTGAAGCCAGGAAAGATT
	R:AGCTCTCTCTTTGACGTTCTGC
FGFR2	F:CCTCCACGGTGGTCGGAG
	R:GGGGGATGCGCTTGGTCAG
HGS	F:GTCAGGGGGACACACAAGCA
	R:GGGCTCTTTCAGCAGCGAAC
hnRNP F 3'UTR M/R	F:GAGTTAAGTAGTTTAAGTATGTTGAGTG
	R:GTCAAGGCCTCACCAATTCCTAC
hnRNP F qPCR M/R	F:GACCACAGATATGGAGACAGCG
	R:GTAGCAAACTCAACATCAGCTTCTC
hnRNP H 3'UTR/qPCR M/R	F:ATTGCATAGGTAGCCAAGGAGCA
	R:GGCATAAAACCACAAGGCTGGAT
hnRNP F 3’UTR H	F:TTGAGAATTGAGACACAATACTAATAC
	R:AGTAATCTGTGCCAGTCATTGTG
hnRNP H 3’UTR H	F:TCATCTAGGATGTAACAGTGAAGC
	R:TGTTCAATTTAACGTGGCAAAGGG
hnRNP H 3’UTR Cloning primers	F:GGATCTCTTTACAGGTAGCCAAG
	R:ACATCAAGTTCACAGGACACTAA
hnRNP H 3’UTR nested cloning primers	F:CGCGCTCGAGGTAATGGAAGCCGAGCATCT
	R:CGCGTCTAGACAAGGTTCACTTAGCGCAATAC
hnRNP H putative QRE mutagenesis primers	F:gagcaaagctgagtgaactgtcctagcttttctctagtta
	R:taactagagaaaagctaggacagttcactcagctttgctc
hnRNP H reporter	F:AAGAGATCGTGGATTACGTCGCCA
	R:GGCATAAAACCACAAGGCTGGAT
MADD	F:TTGCTAGCAGCCTGTATCGGAA
	R:GGTTCTCGCTAGAGTTGCTCG
MAG endogenous specific	F:GTGCTGTGGTCGCCTTTGCC
	R:CCCTCACCCCTACTACTCTC
MAGmini specific	F:GGATCACTCTCGGCATGGAC
	R:GTCTGAGTGGGAATAACTGAGG
MAGmini G-run site #1 mutagenesis primers	F:aggatggcaaggttgctaaacaaaagggaggcc
	R:ggcctcccttttgtttagcaaccttgccatcct
MAGmini G-run site #2 mutagenesis primers	F:ccctattgtgtgtccaaatgctatcaggagtagagagc
	R:gctctctactcctgatagcatttggacacacaataggg
MAST1	F:CCACTTAGGTAGCAGTCCTTTG
	R:CCTTTGGGAACCGCTCCTTG
MBNL2	F:AGGACTGATAAACTGGAGGTATG
	R:TCTTTGGTAAGGGATGAAGAGCA
MPZL1	F:CTCCACGTGGTGGAAATAGACAA
	R:GAGTCCTCAAGGCCACATGC
NF2	F:GAGGAGCCCATGAACCCAATTC
	R:GGAGCAGCAGATGTGGCAGG
UAP1	F:GCAAAGAAGTTTGTGGTGTATG
	R:ATAACCTTCAAGGCCCTCTC
SIRT2	F:CGGGACAGAGCAGTCGGTGA
	R:GGCAGATGGTTGGCTTGAACTG
TARBP2	F:GCGTTTGCCAGAGTACATGGT
	R:CAGTTCCTCAATATCCAGATAGC
TRMU	F:AGGGTGACGTGCTTGTGGCT
	R:CTTACCCTTCTGGAGTGTGTAG
TPM1	F:GGCGGAAGACCGGAGCA
	R:CATGCCTCTCTCACTCTCATCT
Zasp/LBD3	F:ATGACCGGGACAGAATACATGC
	R:GCAGGCACTGGCTGGTAGA
